# Culture-Based Detection of *Campylobacter* spp. in Farmed and Free-Ranging Common Pheasants (*Phasianus colchicus*) from Eastern Poland: Distribution in Intestinal and Edible Tissue Samples and Species Identification by PCR and MALDI-TOF MS

**DOI:** 10.3390/foods15183262

**Published:** 2026-09-15

**Authors:** Monika M. Ziomek, Zbigniew Bełkot, Iga Szostak, Francesca Pedonese, Katarzyna Michalak, Zuzanna J. Strzałkowska, Łukasz Drozd, Joanna Pławińska-Czarnak

**Affiliations:** 1 Department of Food Hygiene of Animal Origin, University of Life Sciences in Lublin, Akademicka 12, 20-950 Lublin, Poland; monika.ziomek@up.edu.pl (M.M.Z.); zbigniew.belkot@up.edu.pl (Z.B.); lukasz.drozd@up.edu.pl (Ł.D.); 2 Student Research Group of Diseases of Game and Free-Living Animals, Faculty of Veterinary Medicine, University of Life Sciences in Lublin, Akademicka 12, 20-033 Lublin, Poland; iszostak@op.pl; 3 Department of Veterinary Sciences, University of Pisa, Viale delle Piagge 2, 56124 Pisa, Italy; francesca.pedonese@unipi.it; 4 Interdepartmental Research Center “Nutraceuticals and Food for Health”, University of Pisa, Via del Borghetto 80, 56124 Pisa, Italy; 5 Department of Epizootiology and Clinic of Infectious Diseases, Faculty of Veterinary Medicine, University of Life Sciences in Lublin, Głęboka 30, 20-612, Lublin, Poland; katarzyna.michalak@up.edu.pl; 6 Department of Preclinical Sciences, Institute of Veterinary Medicine, Warsaw University of Life Sciences-SGGW, Ciszewskiego 8, 02-776 Warsaw, Poland; z.strzalkowska@gmail.com

**Keywords:** foodborne pathogens, game birds, *Phasianus colchicus*, *Campylobacter jejuni*, *Campylobacter coli*, MALDI-TOF MS, multiplex PCR identification

## Abstract

*Campylobacter* spp. are important foodborne pathogens, but data on their occurrence in common pheasants (*Phasianus colchicus*) and their distribution between intestinal and edible tissue samples remain limited. This study aimed to compare the culture-based occurrence of *Campylobacter* spp. in farmed and free-ranging pheasants from eastern Poland and to identify the recovered isolates by multiplex PCR and MALDI-TOF MS. A total of 154 pheasants were examined, including 71 farmed and 83 free-ranging birds. Cloacal swabs, caecal contents, liver tissue, and pectoral muscle samples were collected from each bird, giving 616 samples in total. PCR-confirmed *Campylobacter* isolates were recovered from 20/71 farmed pheasants (28.2%) and from none of the 83 free-ranging pheasants (*p* < 0.001). Among farmed birds, isolates were recovered from 16/71 cloacal swabs (22.5%), 7/71 liver samples (9.9%), and 5/71 pectoral muscle samples (7.0%); no isolates were obtained from caecal contents. Of 28 isolates, 23 (82.1%) were identified as *C. jejuni* and 5 (17.9%) as *C. coli*. MALDI-TOF MS species assignments were concordant with multiplex PCR for all isolates. Under the sampling periods and conditions examined, viable *Campylobacter* was recovered from farmed pheasants, including liver and pectoral muscle samples, whereas no confirmed isolates were obtained from the sampled free-ranging pheasants. Because the two groups differed in sampling period, age and sex structure, and geographical origin, the observed difference cannot be attributed exclusively to husbandry status.

## 1. Introduction

*Campylobacter* spp. remain among the most important bacterial foodborne pathogens worldwide, with *Campylobacter jejuni* and *Campylobacter coli* accounting for the majority of human campylobacteriosis cases. In 2024, campylobacteriosis was the most frequently reported zoonosis in the European Union, with 168,396 confirmed cases and a notification rate of 55.3 cases per 100,000 population [1]. Although poultry meat is recognized as a major vehicle of human exposure, the contribution of less commonly consumed avian species, including game birds, remains less comprehensively characterized [2].

Birds constitute natural reservoirs of *Campylobacter*, and colonized individuals may harbor high bacterial loads without developing evident clinical signs. In chickens, *C. jejuni* preferentially colonizes the caecal mucus and crypts, where it may persist despite the host immune response [3]. Intestinal colonization and faecal shedding facilitate the spread of the organism within flocks and create opportunities for contamination of carcasses and meat during slaughter and processing [4].

The common pheasant (*Phasianus colchicus*) is both a free-ranging game bird and a species maintained under farm or aviary conditions. Pheasant meat may enter the food chain through hunting or specialized game-bird production, while the conditions of killing, transport, evisceration, and carcass handling may be performed under conditions that are less standardized than those applied in industrial poultry production [5]. Game birds may carry zoonotic bacteria in their intestinal tract, creating potential exposure for hunters, persons handling carcasses, and consumers through direct contact, cross-contamination during food preparation, or consumption of inadequately cooked meat [6].

Previous studies have confirmed that pheasants may carry zoonotically relevant *Campylobacter* species, although reported occurrence and species distribution vary considerably. In Scotland, *Campylobacter* was recovered from 36.6% of 287 caecal samples from hunted pheasants, with *C. coli* predominating over *C. jejuni* [7]. In contrast, 72% of intestinal samples collected from hunted reared pheasants in Finland were culture-positive, and all identified isolates belonged to *C. jejuni* [8].

Farmed and free-ranging pheasants are exposed to substantially different management and ecological conditions. In farmed populations, close contact between birds, shared litter, water and feeding facilities, and repeated faecal–oral transmission may promote rapid dissemination following the introduction of *Campylobacter* into a flock [9]. In free-ranging birds, the probability of carriage may instead be influenced by host age, season, habitat use, social behavior, and the frequency of contact with other birds and environmental sources [10].

Simultaneous examination of intestinal and edible tissue samples collected from the same bird may provide a more comprehensive assessment of the potential food safety significance of *Campylobacter* carriage. The caecum represents a principal site of intestinal colonization, whereas examination of caecal contents provides information on intestinal carriage and cloacal swabs provide information on faecal shedding. In contrast, liver and pectoral muscle are edible tissues, and the recovery of *Campylobacter* from these matrices may indicate exposure to contamination during evisceration, carcass handling, or further processing [5,6]. However, culture-based detection in edible tissues alone does not allow ante-mortem bacterial translocation to be distinguished conclusively from post-mortem contamination [11]. A within-bird comparison of these matrices may nevertheless help determine whether intestinal carriage and faecal shedding are accompanied by the recovery of *Campylobacter* from edible tissues.

Culture is essential for demonstrating viable bacteria and recovering isolates for further characterization. However, the fastidious growth requirements of *Campylobacter*, its susceptibility to oxygen exposure and adverse transport conditions, and the limitations of conventional phenotypic identification may affect both bacterial recovery and species assignment [12]. Molecular methods provide reliable species confirmation, whereas MALDI-TOF MS enables rapid and comparatively inexpensive identification of cultured isolates. The method generally performs well for commonly encountered species such as *C. jejuni* and *C. coli*, although identification reliability may be lower for less frequently isolated species because of limited reference-spectrum coverage [13].

Available studies of pheasants and other game birds have generally focused on intestinal carriage, faecal samples, or selected carcass surfaces. Consequently, information remains limited on the within-bird relationship between intestinal carriage, faecal shedding, and the recovery of *Campylobacter* from edible tissues [11]. Although MALDI-TOF MS has been evaluated for the identification of *Campylobacter* isolates from chickens and selected wild bird species, information concerning its agreement with PCR for pheasant-derived isolates remains limited [14].

Therefore, the aim of this study was to compare the culture-based occurrence and matrix-specific distribution of *Campylobacter* spp. in farmed and free-ranging common pheasants (*Phasianus colchicus*) from the Lublin region of Poland. Cloacal swabs, caecal contents, liver tissue, and pectoral muscle samples collected from individual birds were examined to assess intestinal carriage and faecal shedding and to determine the occurrence of *Campylobacter* in edible tissue samples. A further objective was to evaluate the agreement between PCR and MALDI-TOF MS in the species-level identification of the recovered isolates.

## 2. Materials and Methods

### 2.1. Study Design, Bird Population, and Husbandry Conditions

A cross-sectional study was conducted on 154 common pheasants (*Phasianus colchicus*) originating from the Lublin region of eastern Poland. The study population comprised 71 farmed pheasants obtained from three farms and 83 free-ranging pheasants harvested during regular hunting activities in 11 hunting grounds.

The investigated farms differed in the annual number of pheasants reared. Farm A reared approximately 500 pheasants per year, Farm B approximately 2000 birds per year, and Farm C approximately 5000 birds per year. All three farms used an aviary-based husbandry system, in which pheasants were maintained in fenced aviaries with year-round access to outdoor runs. Stocking density varied according to bird age and ranged from approximately 1–2 birds/m^2^ during the growing period to approximately one adult bird per 3–4 m^2^. Although mesh fencing physically separated the farmed pheasants from free-ranging birds, year-round outdoor access did not completely exclude potential exposure to wild birds and environmental sources of microorganisms.

The production system was primarily oriented towards rearing pheasants for subsequent release. Approximately 10% of the birds were retained as breeding stock, whereas the remaining birds were reared until they reached an appropriate stage of development and subsequently released into hunting grounds in late summer or autumn, as part of local game-management practices aimed at supplementing free-ranging pheasant populations.

The farmed group consisted of 40 females and 31 males aged 6–13 months. The numbers of birds originating from Farms A, B, and C were 20, 25, and 26, respectively. Farmed pheasants were sampled between October 2022 and January 2024. The age of farmed pheasants was provided by the farm owners.

The free-ranging group consisted of 82 males and one female, with recorded age estimates ranging from 5 to 48 months. These birds were harvested between December 2022 and February 2023. In free-ranging males, age class was estimated in the field by an experienced hunter with approximately 30 years of hunting experience, based on a combination of external morphological characteristics. Particular attention was paid to spur characteristics, including length, shape, sharpness or bluntness, color, hardness, surface texture, and degree of wear, while plumage and flight-feather characteristics were considered as additional age indicators [15,16,17]. Qualitative and quantitative spur characteristics have traditionally been used to distinguish juvenile from adult male pheasants; however, overlap between age classes and variation associated with the timing of the hunting season and population of origin may limit the accuracy of estimates based on spur length alone [15,16]. Therefore, morphological assessment was used primarily for assignment to age classes, whereas age values expressed in months represented approximate field estimates rather than exact determinations of chronological age. For the single free-ranging female, the age value recorded at collection was retained as an approximate field estimate.

Individual demographic characteristics, anonymized origin, sampling dates, and intervals between slaughter or hunting and laboratory examination are provided in Tables S2 and S3. Aggregated characteristics of the study population and sampling design are presented in Table S1. Because the farmed and free-ranging groups differed in sampling period, sex and age structure, and geographical origin, the comparison was observational and was not designed to isolate husbandry status as an independent causal factor.

### 2.2. Sample Collection and Transport

Following slaughter or hunting, pheasant carcasses were stored at 4 °C and transported to the laboratory under refrigerated conditions. Laboratory examination and sample collection were performed 16–24 h after slaughter in farmed pheasants and 16–36 h after hunting in free-ranging pheasants. To minimize the risk of faecal cross-contamination, samples were collected in a predefined order: pectoral muscle tissue, liver tissue, caecal contents, and finally a cloacal swab. Separate sterile instruments were used for each sample type. The superficial tissue layer of the liver and pectoral muscle was aseptically removed with sterile instruments, and samples for microbiological examination were collected from the underlying internal tissue. No chemical or thermal surface decontamination was applied before sampling. Immediately after collection, all samples were subjected to selective enrichment in Preston broth as described in Section 2.3. A total of 616 samples were analyzed, including 284 samples collected from farmed pheasants and 332 samples from free-ranging pheasants.

### 2.3. Campylobacter Enrichment and Isolation

Detection of *Campylobacter* spp. was performed using a modified Procedure B of PN-EN ISO 10272-1:2017 [18]. Immediately after collection, liver and pectoral muscle samples were transferred to Preston enrichment broth prepared using Preston Broth Base (PS 255) supplemented with Preston Modified Supplement (SL 0084), Preston *Campylobacter* Growth Supplement (SL 0149), and Lysed Horse Blood (SL 0103) (BioMaxima, Lublin, Poland), using 10 g of tissue and 90 mL of broth. For caecal-content samples, 1 g of luminal contents collected from a single caecum was added to 9 mL of the same enrichment broth. The paired caeca were not pooled, and the caecal mucosa was not sampled separately. Cloacal swabs were placed directly into sterile tubes containing 5 mL of Preston broth. All samples were vortexed for 30 s. The enrichment cultures were incubated for 48 h at 41.5 ± 0.5 °C under microaerobic conditions (5% O_2_, 10% CO_2_, and 85% N_2_). The 48 h enrichment period was selected after preliminary optimization of the culture procedure, during which subculture after 24 h of Preston enrichment did not yield presumptive *Campylobacter* colonies, whereas extension of enrichment to 48 h enabled recovery of PCR-confirmed *Campylobacter*. The 48 h enrichment period was also consistent with previously published Preston broth protocols used for poultry caecal, faecal, and meat samples [19,20,21] and represents a modification of the 24 ± 2 h incubation specified in ISO 10272-1:2017 Procedure B [18]. Following enrichment, the cultures were streaked onto mCCDA agar (BioMaxima, Lublin, Poland) and incubated for 44 ± 4 h at 41.5 ± 0.5 °C under microaerobic conditions.

### 2.4. Preliminary Phenotypic Identification and Isolate Storage

Presumptive *Campylobacter* colonies were initially selected on the basis of colony morphology on mCCDA agar. For each sample showing presumptive growth, one representative colony was selected for further examination and subcultured on Columbia Blood Agar (BioMaxima, Lublin, Poland) for 24 h at 41.5 ± 0.5 °C under microaerobic conditions.

Following incubation, colonies were tested for oxidase and catalase activity using Bactident^®^ Oxidase strips (Merck KGaA, Darmstadt, Germany) and 3% hydrogen peroxide solution (Merck KGaA, Darmstadt, Germany), respectively. The presumptive isolates were then cultured on CHROMagar™ *Campylobacter* (CHROMagar, Paris, France) and incubated for 48 h at 41.5 ± 0.5 °C under microaerobic conditions. After incubation, one representative colony from each CHROMagar plate was subsequently subcultured on Columbia Blood Agar and incubated for 24 h at 41.5 ± 0.5 °C under microaerobic conditions to obtain a pure culture. All purified presumptive isolates, irrespective of colony morphology, were stored at −86 °C using the Cryobank™ bacterial culture freezing system (COPAN Diagnostics, Brescia, Italy) until further analyses. Multiplex PCR was subsequently performed as described in Section 2.5. PCR-confirmed *Campylobacter* isolates were further subjected to MALDI-TOF MS analysis.

### 2.5. DNA Extraction and Multiplex PCR Identification

Genomic DNA was extracted from purified bacterial cultures using NucleoSpin Microbial DNA (Macherey-Nagel, Dueren, Germany) in accordance with the manufacturer’s protocol for Gram-negative bacteria. DNA extracts were stored at −18 °C until amplification.

Species-level identification of the isolates was performed using a multiplex PCR assay based on the method described by Seliwiorstow et al., with modifications to template preparation, reaction mixture composition, and thermal cycling conditions, as detailed below [22]. The assay enables differentiation of *Campylobacter jejuni*, *Campylobacter coli*, *Campylobacter lari*, *Campylobacter upsaliensis*, and *Campylobacter fetus* subsp. *fetus*. Purified genomic DNA obtained from the bacterial isolates was used as the template for amplification. Compared with the original protocol of Wang et al. [22], purified genomic DNA was used instead of a whole-cell template, a commercial 2× PCR master mix was applied, and the thermal cycling conditions were modified as described below.

Each PCR reaction was performed in a final volume of 25 µL containing 12.5 µL of oasig^®^ Lyophilised 2× qPCR Master Mix (Primerdesign Ltd., Chandler’s Ford, Southampton, UK), 0.5 µL of each of the 12 primers (6.0 µL total primer volume), 2.5 µL of genomic DNA, and 4.0 µL of nuclease-free water. Primer working solutions were prepared from 100 µM stock solutions (Genomed, Warsaw, Poland). The working concentrations were adjusted to obtain final primer concentrations of 0.2 µM for 23SF/23SR, 0.5 µM for CJF/CJR and CLF/CLR, 1.0 µM for CCF/CCR and CFF/CFR, and 2.0 µM for CUF/CUR, in accordance with the multiplex PCR scheme described by Wang et al. Primer working concentrations, sequences, target genes, reaction volumes, final concentrations, and expected amplicon sizes are provided in Supplementary Table S4.

PCR amplification was performed using the following thermal cycling conditions: an initial denaturation step at 94 °C for 4 min, followed by 30 amplification cycles. Each cycle consisted of denaturation at 94 °C for 1 min primer annealing at 58 °C for 1 min, and extension at 72 °C for 2 min. The amplification was completed with a final extension step at 72 °C for 5 min.

Genomic DNA from *Campylobacter jejuni* ATCC 33560™, *Campylobacter coli* ATCC 33559™, *Campylobacter lari* ATCC 35221™, *Campylobacter upsaliensis* ATCC 43954™, and *Campylobacter fetus* subsp. *fetus* ATCC 27374™ (American Type Culture Collection, Manassas, VA, USA) was used as positive controls. Nuclease-free water was included as a no-template control.

The amplification products were separated by horizontal electrophoresis in 2% agarose gels prepared in TBE electrophoresis buffer. Electrophoresis was conducted at 90 V for 75 min. A 100–1000 bp ladder (A&A Biotechnology, Gdańsk, Poland) was used as a molecular mass marker. DNA fragments were stained with SimplySafe (Eurx, Gdańsk, Poland) and visualized using a Vilber Quantum gel documentation system (Vilber Lourmat, Collégien, France).

Species identification was based on the presence of species-specific amplification products of the expected sizes described by Wang et al. [22]. The 650 bp 23S rRNA amplicon generated by the 23SF/23SR primer pair served as an internal validation control, as described by Wang et al. [22], and was not interpreted as a *Campylobacter* genus-specific marker. An isolate was assigned to a given *Campylobacter* species when both the 650 bp internal validation product and the corresponding species-specific amplicon were present in the sample lane, the appropriate positive control yielded the expected amplification products, and no amplification was observed in the no-template control (Supplementary Table S4).

### 2.6. MALDI-TOF MS Identification

All PCR-confirmed isolates were further subjected to species-level identification by matrix-assisted laser desorption/ionization–time-of-flight mass spectrometry. Protein extraction was performed using the ethanol/formic acid extraction procedure.

Briefly, a single colony was suspended in 150 µL of sterile deionized water, followed by the addition of 450 µL of absolute ethanol. The suspension was mixed thoroughly and centrifuged for 5 min at 13,000 rpm. After removal of the supernatant, the pellet was resuspended in 40 µL of 70% formic acid and 40 µL of acetonitrile. The suspension was vortexed and centrifuged again for 5 min at 13,000 rpm.

One microliter of the resulting supernatant was applied in triplicate to a reusable steel target plate and allowed to dry at room temperature. After drying, each spot was overlaid with 1 µL of α-cyano-4-hydroxycinnamic acid (HCCA) matrix solution.

Mass spectra were acquired using an UltrafleXtreme mass spectrometer and analyzed using MALDI Biotyper software version 3.1 with the MALDI Biotyper reference database V09.0.0.0_8468, containing 8468 reference profiles (Bruker Daltonik, Bremen, Germany). Spectra were recorded in the m/z range of 2000–20,000 using 2000 laser shots per spot. The highest MALDI Biotyper score obtained from the three technical measurements was used for the final isolate-level identification. MALDI Biotyper score values of 2.300–3.000 were interpreted as highly probable species-level identification, values of 2.000–2.299 as secure genus-level and probable species-level identification, values of 1.700–1.999 as probable genus-level identification, and values below 1.700 as unreliable identification.

### 2.7. Statistical Analysis

Differences in the proportion of birds yielding PCR-confirmed *Campylobacter* isolates between farmed and free-ranging pheasants were assessed using the two-sided Fisher’s exact test, with the bird considered the primary statistical unit. A bird was classified as positive when at least one of the four examined samples yielded a culture isolate confirmed as *Campylobacter* by multiplex PCR.

Matrix-specific differences between farmed and free-ranging pheasants were evaluated separately using two-sided Fisher’s exact tests. Because four matrix-specific comparisons were performed, the resulting *p*-values were adjusted using the Holm method to control the family-wise error rate. Sample-level proportions calculated across all four matrices were reported descriptively because samples collected from the same bird were not statistically independent. A two-sided *p*-value below 0.05 was considered statistically significant for the primary bird-level comparison. For matrix-specific comparisons, Holm-adjusted *p*-values below 0.05 were considered statistically significant. Statistical analyses were performed using GraphPad Prism version 7.0 (GraphPad Software, La Jolla, CA, USA).

## 3. Results

### 3.1. Overall Occurrence of PCR-Confirmed Campylobacter spp.

A total of 154 common pheasants and 616 samples were examined. Culture-based examination yielded 49 presumptive isolates, all of which were subjected to multiplex PCR. Twenty-eight isolates were PCR-confirmed as *Campylobacter*, whereas the remaining 21 presumptive isolates yielded no amplification product, including the 650 bp 23S rRNA internal validation product. No presumptive isolate yielded the 650 bp internal validation product in the absence of a species-specific amplicon. PCR-confirmed *Campylobacter* isolates were recovered from 20 of the 71 farmed pheasants (28.2%), whereas none of the 83 free-ranging pheasants yielded a confirmed isolate. The proportion of birds yielding PCR-confirmed *Campylobacter* isolates was significantly higher among farmed than free-ranging pheasants (two-sided Fisher’s exact test, *p* < 0.001).

At the sample level, PCR-confirmed isolates were recovered from 28 of the 284 samples collected from farmed pheasants (9.9%), whereas none of the 332 samples obtained from free-ranging pheasants yielded a confirmed isolate. Overall, *Campylobacter* was confirmed in 20 of the 154 examined birds (13.0%) and in 28 of the 616 examined samples (4.5%). Because four samples were collected from each bird, sample-level proportions were treated as descriptive results. The occurrence of PCR-confirmed *Campylobacter* at the bird and sample levels is summarized in Table 1.

### 3.2. Matrix-Specific Distribution of Campylobacter spp.

PCR-confirmed *Campylobacter* isolates were recovered from three of the four sample matrices collected from farmed pheasants. The highest recovery frequency was recorded for cloacal swabs, from which confirmed isolates were obtained in 16 of 71 birds (22.5%). Isolates were also recovered from liver tissue in 7 of 71 birds (9.9%) and from pectoral muscle tissue in 5 of 71 birds (7.0%). No PCR-confirmed isolates were recovered from caecal contents. None of the corresponding matrices collected from the 83 free-ranging pheasants yielded a confirmed isolate. Matrix-specific results are presented in Table 2.

After adjustment for four matrix-specific comparisons using the Holm method, the differences between farmed and free-ranging pheasants remained statistically significant for cloacal swabs (adjusted *p* < 0.001), liver tissue (adjusted *p* = 0.0112), and pectoral muscle tissue (adjusted *p* = 0.0385). No difference could be demonstrated for caecal contents because no PCR-confirmed isolates were recovered from either group.

PCR-confirmed *Campylobacter* isolates were recovered from pheasants originating from all three farms. The farm-specific proportion of positive birds was 20.0% on Farm A (4/20), 32.0% on Farm B (8/25), and 30.8% on Farm C (8/26). These birds yielded 5, 10, and 13 isolates, respectively. Overall, 20 of the 71 farmed pheasants were positive (28.2%), yielding a total of 28 isolates. Farm-specific results are summarized in Table 3, whereas the individual positive birds and their corresponding isolates are listed in Supplementary Table S5. The recovery of isolates from all three farms indicates that positivity was not confined to a single farm.

### 3.3. Individual-Level and Species-Specific Distribution of PCR-Confirmed Campylobacter Isolates

Among the 20 farmed pheasants yielding PCR-confirmed *Campylobacter* isolates, 13 birds yielded an isolate from one of the four examined matrices, six birds yielded isolates from two matrices, and one bird yielded isolates from three matrices. Concurrent recovery from a cloacal swab and liver tissue was observed in three birds, whereas concurrent recovery from a cloacal swab and pectoral muscle tissue was recorded in two birds. One bird yielded isolates from both liver and pectoral muscle tissue, and one bird yielded isolates from the cloacal swab, liver tissue, and pectoral muscle tissue. No PCR-confirmed isolate was recovered from caecal contents, either alone or in combination with another matrix.

Multiplex PCR identified 23 of the 28 representative isolates (82.1%) as *Campylobacter jejuni* and 5 (17.9%) as *Campylobacter coli*. Of the 16 isolates recovered from cloacal swabs, 11 (68.8%) were identified as *C. jejuni* and 5 (31.2%) as *C. coli*. All seven isolates recovered from liver tissue and all five isolates recovered from pectoral muscle tissue were identified as *C. jejuni*. Among the representative isolates characterized, *C. coli* was recovered only from cloacal swabs, whereas *C. jejuni* was recovered from all three matrices yielding confirmed isolates.

The *C. jejuni* isolates originated from 15 farmed pheasants, whereas *C. coli* was recovered from five farmed pheasants. In birds yielding representative isolates from more than one matrix, species identification was concordant across all characterized isolates. Among the representative isolates examined, no bird yielded isolates identified as both *C. jejuni* and *C. coli*. However, because only one representative isolate was characterized from each positive sample, mixed-species populations within individual samples or birds cannot be excluded. No other *Campylobacter* species included in the multiplex PCR assay were identified among the representative isolates. Multiplex PCR amplification profiles for all 28 isolates, together with the reference strains and no-template control, are presented in Supplementary Figure S1. Individual bird-, matrix-, and isolate-specific PCR results are provided in Supplementary Table S5.

### 3.4. MALDI-TOF MS Identification and Agreement with Multiplex PCR

All 28 PCR-confirmed isolates were additionally analyzed by MALDI-TOF MS. The species assignments obtained by MALDI-TOF MS were concordant with the multiplex PCR results for all examined isolates. Thus, 23 isolates were assigned to *C. jejuni* and 5 isolates to *C. coli* by both identification methods.

MALDI-TOF MS confidence scores varied among technical measurements; however, the best-match species assignments were consistent with the corresponding PCR identifications. Individual MALDI-TOF MS identifications and score values are presented in Supplementary Table S5.

## 4. Discussion

The present study identified a significant difference between farmed and free-ranging common pheasants in the proportion of birds yielding culture isolates of *Campylobacter* spp. subsequently confirmed by multiplex PCR. Confirmed isolates were obtained from 28.2% of farmed pheasants, whereas none of the samples collected from the 83 free-ranging birds yielded a confirmed isolate. Positive birds originated from all three investigated farms, with farm-specific proportions ranging from 20.0% to 32.0%, indicating that the overall result was not attributable to the occurrence of *Campylobacter* in a single farm. Importantly, the farms differed substantially in annual production scale, rearing approximately 500, 2000, and 5000 pheasants per year. However, because only 20–26 birds were examined per farm and the study was not designed to evaluate farm-level risk factors, no association between farm size and *Campylobacter* occurrence can be inferred. The proportion observed in the present study was lower than the 72% culture positivity reported for intestinal samples from hunted reared pheasants in Finland, but it was comparable to the 36.6% positivity recorded in caecal samples from game pheasants in Scotland [7,8]. Direct comparisons between studies should nevertheless be made cautiously because of differences in bird origin and age, management conditions, sampling period, sample matrix, and microbiological methodology.

The recovery of confirmed isolates from all three farms suggests that the occurrence of *Campylobacter* in the investigated farmed population was not associated with a single localized event. Management conditions such as close contact between birds, shared drinkers and feeders, and exposure to faecally contaminated litter may facilitate faecal–oral transmission following the introduction of the organism into a flock. Kivistö et al. demonstrated the occurrence of two *C. jejuni* clones during successive samplings of an intensively managed pheasant flock, with one clone persisting for at least one month [8]. A longitudinal investigation of broiler farms also detected genetically related isolates in faeces, litter, drinkers, barn equipment, and the surrounding farm environment [23]. Although observations from commercial broiler production cannot be extrapolated directly to pheasant farming, they support the potential role of contaminated litter, water, and equipment in the circulation of *Campylobacter* within managed flocks. In the present study, general stocking densities ranged from approximately 1–2 birds/m^2^ during the growing period to one adult bird per 3–4 m^2^. However, pen-specific stocking density at the time of sampling, water quality, litter conditions, biosecurity practices, and environmental contamination were not systematically assessed. Therefore, no individual husbandry factor can be identified as the source of the observed occurrence.

The absence of confirmed isolates among the 83 free-ranging pheasants contrasts with the results of several previous European investigations. In Finland, *Campylobacter* DNA was detected in 21.8% of faecal samples from pheasants obtained during the hunting season, whereas viable organisms were recovered from 36.6% of caecal samples collected from game pheasants in Scotland [6,7]. Consequently, the result of 0/83 should not be interpreted as evidence that free-ranging pheasants are generally free from *Campylobacter*. Rather, it characterizes the populations sampled in the investigated hunting grounds during the specified period and under the culture conditions applied. The husbandry model used on the investigated farms is also relevant in this context because approximately 90% of the reared pheasants were ultimately intended for release into hunting grounds. Thus, managed and free-ranging pheasant populations may be connected through routine game-management practices rather than representing completely isolated epidemiological compartments. The present study, however, did not follow released individuals or investigate matched environmental samples and therefore provides no evidence for transmission of *Campylobacter* from farmed to free-ranging pheasants or in the opposite direction. Differences between studies may reflect geographical and environmental variation, season, bird age, access to surface water, contact with livestock or other birds, and differences in sample collection, storage, and detection methodology. Moreover, the farmed and free-ranging groups examined in the present study differed in sex and age structure, sampling period, and geographical origin. The observed difference therefore cannot be attributed exclusively to farmed versus free-ranging status.

Cloacal swabs showed the highest matrix-specific recovery among farmed pheasants, whereas no PCR-confirmed isolates were obtained from caecal contents. This unexpected discordance may reflect both biological heterogeneity and methodological factors. Only 1 g of luminal contents from a single caecum was examined from each bird, and the paired caeca were not pooled. *Campylobacter* may be unevenly distributed within caecal material and preferentially associated with the mucus layer and caecal crypts rather than uniformly present in the luminal contents [3]. Consequently, examination of luminal contents from only one caecum may have reduced the probability of recovery. Culture recovery may additionally have been influenced by bacterial concentration, competing microbiota, selective media, enrichment procedures, and storage conditions [24,25]. The Preston broth enrichment period was extended to 48 h following preliminary optimization of the culture procedure and was also consistent with previous poultry studies using 48 h enrichment [19,20,21]. Because this differed from the 24 ± 2 h incubation specified in ISO 10272-1:2017 Procedure B, an effect of the modified enrichment conditions on recovery cannot be excluded [18]. Therefore, the absence of confirmed isolates from caecal contents should be interpreted cautiously and does not exclude intestinal colonization.

The recovery of viable *Campylobacter* from liver and pectoral muscle samples is relevant to food safety because both tissues may be intended for human consumption. However, these findings require cautious interpretation. Although the superficial tissue layer was aseptically removed and samples were collected from the underlying tissue using sterile instruments, no chemical or thermal surface decontamination was applied before sampling. Therefore, superficial contamination or carry-over during sample preparation cannot be completely distinguished from the presence of bacteria in deeper tissue. It is therefore not possible to determine whether the organisms were located within the tissue or were introduced from its surface during slaughter, evisceration, transport, or sampling. Berrang et al. detected *Campylobacter* on both the surface and within the internal tissue of broiler livers, while subtype analysis indicated that isolates from the caeca and liver could represent either identical or different subtypes [26]. Luber and Bartelt detected the organism on the surface of 87% of examined chicken breast fillets and within the deeper tissue of 20%, although internal bacterial concentrations were very low [27]. The present findings should therefore be described as recovery of *Campylobacter* from liver and pectoral muscle samples rather than as evidence of systemic infection, bacterial translocation, or confirmed internal tissue contamination.

The recovery of viable *Campylobacter* from liver and pectoral muscle samples is of direct relevance to food safety because these tissues may enter the food chain as edible offal and meat, respectively. From a hazard analysis perspective, their contamination is important not only because of the potential for direct consumer exposure following insufficient heat treatment, but also because contaminated raw tissues may facilitate bacterial dissemination during evisceration, carcass dressing, portioning, meat preparation, and contact with food-handling equipment. Edible tissues carrying viable bacteria may serve as sources of contamination for hands, knives, cutting boards, containers, and other foods, including products that will not undergo further heat treatment.

Recent investigations of broiler livers demonstrated the presence of *Campylobacter* on both the organ surface and within internal liver tissue. Surface contamination was more frequent and generally occurred at higher bacterial loads, although viable bacteria were also detected within the tissue, which is particularly relevant when liver products are served undercooked or with an incompletely heated interior [28]. Studies conducted in domestic kitchens have also confirmed the transfer of *Campylobacter* from naturally contaminated poultry meat to cutting boards, sinks, and kitchen cloths. Importantly, cross-contamination events were documented even during the preparation of meat carrying relatively low bacterial loads [29]. These observations indicate that the food safety consequences of contaminated edible tissues are not restricted to direct consumption, as secondary transfer to ready-to-eat foods and food-contact surfaces may also contribute to consumer exposure.

In the present study, the superficial tissue layer of the liver and pectoral muscle was aseptically removed before samples were collected from the underlying tissue; however, no chemical or thermal surface decontamination was applied. Consequently, it is not possible to determine conclusively whether the recovered isolates represented bacteria present in deeper tissue or residual contamination introduced during slaughter, evisceration, transport, or sample preparation. Nevertheless, viable *Campylobacter* was recovered from edible-tissue samples, indicating a potential contamination point within the food chain. As bacterial concentrations were not determined and no exposure assessment or evaluation of cooking efficacy was performed, the study identifies a potential microbiological hazard but does not allow the probability or magnitude of consumer risk to be estimated.

Seven farmed pheasants yielded confirmed isolates from more than one sample matrix, including one bird from which isolates were recovered concurrently from the cloacal swab, liver, and pectoral muscle. Among the representative isolates characterized, isolates obtained from different positive matrices within the same bird were assigned to the same species. This pattern is compatible with a common source within the individual carcass, including contamination of tissues with gastrointestinal contents during post-mortem handling. However, species-level concordance does not demonstrate that the isolates were genetically identical. Berrang et al. showed that isolates obtained from the caeca, liver surface, and internal liver tissue of the same broiler carcass could represent either the same or different subtypes [26]. Kivistö et al. similarly identified two distinct *C. jejuni* clones within a single pheasant flock using whole-genome sequencing [8]. MLST, cgMLST, or whole-genome sequencing would therefore be required to determine whether isolates recovered from different matrices of the same pheasant represented the same strain.

Among the representative isolates characterized, *Campylobacter jejuni* predominated, accounting for 82.1%, whereas *C. coli* represented 17.9%. This distribution resembles the findings from Finland, where all pheasant-derived isolates were identified as *C. jejuni* [8]. A contrasting species profile was reported in Scotland, where *C. coli* accounted for 62.6% and *C. jejuni* for 37.4% of PCR-identified isolates [7]. These differences indicate that the species composition of *Campylobacter* populations associated with pheasants may vary according to geographical origin, management conditions, local environmental sources, and the characteristics of the sampled population. Among the representative isolates characterized, *C. coli* was identified only in isolates recovered from cloacal swabs, whereas representative isolates recovered from liver and pectoral muscle samples were identified as *C. jejuni*. Because only one representative isolate was characterized from each positive sample, mixed-species populations within individual samples cannot be excluded. Given the small number of *C. coli* isolates, this pattern should be regarded as descriptive and should not be interpreted as evidence of a greater ability of *C. jejuni* to contaminate or colonize edible tissues.

Species assignments obtained by MALDI-TOF MS were concordant with multiplex PCR for all 28 isolates, supporting the usefulness of MALDI-TOF MS as a rapid complementary approach for the identification of cultured *Campylobacter* isolates. Lawton et al. reported complete agreement between MALDI-TOF MS and the combined interpretation of 16S rDNA sequencing and *hipO* PCR for 27 isolates from poultry and wild birds; complete agreement with WGS was also obtained for all 14 sequenced isolates [14]. Full concordance between MALDI-TOF MS and multiplex PCR was likewise reported for Polish *C. jejuni* and *C. coli* isolates from broilers and turkeys [30]. In the present study, however, score values varied between technical measurements, and not every individual measurement reached the conventional threshold for probable species-level identification. The findings should therefore be interpreted as concordance of the final species assignments rather than uniformly high-confidence identification in every technical replicate. Moreover, MALDI-TOF MS does not replace strain-level molecular typing in epidemiological investigations.

The recovery of viable *C. jejuni* and *C. coli* from pheasants is relevant to farmers, veterinarians, hunters, slaughter and evisceration personnel, meat producers, and consumers. Contact with faeces and viscera may result in occupational exposure, whereas the presence of bacteria in edible tissue samples creates opportunities for transfer to hands, knives, working surfaces, and other foods. Sauvala et al. demonstrated the presence of foodborne pathogens in the faeces of hunted game birds and emphasized the importance of hygienic evisceration, transport, and carcass preparation [6]. Cardoso et al. confirmed the transfer of *Campylobacter* from naturally contaminated poultry meat to cutting boards, sinks, and kitchen cloths under domestic food-preparation conditions [28]. However, the present study did not determine bacterial concentrations, virulence-associated traits, antimicrobial susceptibility, or genomic relatedness to human clinical isolates. The findings therefore identify a potential biological hazard but do not constitute a quantitative assessment of consumer risk.

A major strength of the present study was the collection of four different matrices from each bird, enabling the concurrent evaluation of bacterial shedding and occurrence in edible-tissue samples at the individual-bird level. Additional strengths included culture-based demonstration of bacterial viability and independent species confirmation by multiplex PCR and MALDI-TOF MS. Several limitations should nevertheless be acknowledged. Culture-based detection may have failed to identify injured or viable but non-culturable cells. Okada et al. showed that, although the overall detection frequencies obtained by culture and PMA-qPCR in poultry meat were similar, individual samples were not always classified consistently by the two approaches [31]. In addition, the pheasant groups differed in demographic structure, sampling period, and geographical origin; birds were clustered within farms and hunting grounds; caecal contents were collected from only one caecum per bird and paired caeca were not pooled; no chemical or thermal surface decontamination was applied to liver and pectoral muscle before sampling; bacterial concentrations were not determined; and only one representative isolate from each positive sample was characterized. Future investigations should combine culture with quantitative detection, distinguish surface from deep-tissue contamination, and apply whole-genome sequencing. The value of WGS in pheasant studies was demonstrated by Kivistö et al., who differentiated two *C. jejuni* clones and compared them with isolates originating from humans, animals, and environmental sources [8].

## 5. Conclusions

Farmed pheasant production may facilitate the persistence of *Campylobacter* and increase the likelihood of its transfer to edible tissues. A key unresolved issue is whether the presence of these bacteria in tissues results from colonization of the birds or from secondary contamination of the carcasses. Further studies should include whole-genome sequencing of the isolates and phenotypic antimicrobial susceptibility testing to determine strain relatedness, identify potential transmission routes, and assess their significance as a reservoir of antimicrobial resistance.

## Figures and Tables

**Table 1:** Occurrence of PCR-confirmed *Campylobacter* spp. in farmed and free-ranging common pheasants (*Phasianus colchicus*) at the bird and sample levels.

Parameter	Farmed Pheasants	Free-Ranging Pheasants	Total
Birds examined, *N*	71	83	154
Birds yielding confirmed *Campylobacter* isolates, *n*/*N* (%)	20/71 (28.2)	0/83 (0.0)	20/154 (13.0)
Samples examined, *N*	284	332	616
Samples yielding confirmed *Campylobacter* isolates, *n*/*N* (%)	28/284 (9.9)	0/332 (0.0)	28/616 (4.5)

*N*, total number of birds or samples examined; *n*, number of birds or samples yielding a culture isolate confirmed as *Campylobacter* by multiplex PCR. A bird was classified as positive when at least one of the four samples collected from that individual yielded a PCR-confirmed *Campylobacter* isolate.

**Table 2:** Matrix-specific recovery of PCR-confirmed *Campylobacter* isolates from farmed and free-ranging common pheasants (*Phasianus colchicus*).

Sample Matrix	Farmed Pheasants, *n*/*N* (%)	Free-Ranging Pheasants, *n*/*N* (%)	Raw *p*-Value	Holm-Adjusted *p*-Value
Cloacal swab	16/71 (22.5)	0/83 (0.0)	<0.001	<0.001
Liver tissue	7/71 (9.9)	0/83 (0.0)	0.0037	0.0112
Pectoral muscle tissue	5/71 (7.0)	0/83 (0.0)	0.0193	0.0385
Caecal contents	0/71 (0.0)	0/83 (0.0)	1	1

*N*, number of matrix-specific samples examined; *n*, number of samples yielding a culture isolate confirmed as *Campylobacter* by multiplex PCR. Raw *p*-values were calculated using two-sided Fisher’s exact tests. Adjusted *p*-values were obtained using the Holm method for four matrix-specific comparisons.

**Table 3:** Farm-specific occurrence of PCR-confirmed *Campylobacter* isolates in farmed common pheasants.

Farm	Positive Birds, *n*	Birds Examined, *N*	Positive Birds, *n*/*N* (%)	Number of Isolates
A	4	20	4/20 (20.0)	5
B	8	25	8/25 (32.0)	10
C	8	26	8/26 (30.8)	13
Total	20	71	20/71 (28.2)	28

*N*, total number of pheasants examined on each farm; *n*, number of birds yielding at least one culture isolate confirmed as *Campylobacter* by multiplex PCR.

## Data Availability

The data presented in this study are available within the article and its Supplementary Materials. Additional data are available from the corresponding author upon reasonable request.

## Supplementary Materials

The following supporting information can be downloaded at: https://www.mdpi.com/article/10.3390/foods15183262/s1, Table S1: Summary characteristics of the study population and sampling design; Table S2: Individual characteristics and sampling schedule of farmed common pheasants (*Phasianus colchicus*) (*n* = 71); Table S3: Individual characteristics and sampling schedule of free-ranging common pheasants (*Phasianus colchicus*) (*n* = 83); Table S4: Primers and reaction concentrations used in the multiplex PCR assay for identification of *Campylobacter* isolates; Table S5: Individual multiplex PCR and MALDI-TOF MS identification results for Campylobacter isolates recovered from farmed common pheasants; Figure S1: Multiplex PCR identification of *Campylobacter* isolates according to the method of Wang et al. [22]. M, 100-1000 bp DNA molecular size marker. Arrows indicate the expected sizes of the genus-associated 23S rRNA amplicon (650 bp) and species-specific amplification products. Upper panel, lanes 1-18: isolates 1-13FK, 2-13FW, 3-15FK, 4-16FP, 5-17FK, 6-32FK, 7-32FW, 8-33FK, 9-34FK, 10-34FW, 11-37FK, 12-38FK, 13-38FP, 14-39FP, 15-39FW, 16-40FK, 17-40FP, and 18-40FW, respectively. Isolates 17FK (lane 5) and 37FK (lane 11) were identified as *C. coli*, based on the presence of the 650-bp 23S rRNA amplicon and the species-specific 126-bp *glyA* amplicon. The remaining isolates in the upper panel were identified as *C. jejuni*, showing the 650-bp 23S rRNA amplicon together with the species-specific 323-bp *hipO* amplicon. Lower panel, lanes 19-28: isolates 19-50FK, 20-51FK, 21-53FK, 22-54FK, 23-56FK, 24-60FW, 25-61FW, 26-64FK, 27-64FP, and 28-70FK, respectively. Isolates 53FK (lane 21), 56FK (lane 23), and 70FK (lane 28) were identified as *C. coli*, whereas the remaining isolates were identified as *C. jejuni*. NTC, no-template control. CC, *C. coli* ATCC 33559™; CU, *C. upsaliensis* ATCC 43954™; CL, *C. lari* ATCC 35221™; CJ, *C. jejuni* ATCC 33560™; CFF, *C. fetus* subsp. *fetus* ATCC 27374™. The reference strains produced the expected species-specific amplicons of 126 bp (*C. coli*), 204 bp (*C. upsaliensis*), 251 bp (*C. lari*), 323 bp (*C. jejuni*), and 435 bp (*C. fetus* subsp. *fetus*), together with the 650-bp 23S rRNA amplification product.

## Abbreviations

The following abbreviations are used in this manuscript:

ATCC	American Type Culture Collection
CFF/CFR	*Campylobacter fetus* subsp. *fetus* forward/reverse primers
CCF/CCR	*Campylobacter coli* forward/reverse primers
CJF/CJR	*Campylobacter jejuni* forward/reverse primers
CLF/CLR	*Campylobacter lari* forward/reverse primers
CUF/CUR	*Campylobacter upsaliensis* forward/reverse primers
HCCA	α-cyano-4-hydroxycinnamic acid
MALDI-TOF MS	matrix-assisted laser desorption/ionization–time-of-flight mass spectrometry
mCCDA	modified charcoal cefoperazone deoxycholate agar
NTC	no-template control
PCR	polymerase chain reaction
TBE	Tris–borate–EDTA buffer
23SF/23SR	23S rRNA forward/reverse primers
WGS	whole-genome sequencing
