# Supplementary material for: Culture-Based Detection of Campylobacter spp. in Farmed and Free-Ranging Common Pheasants (Phasianus colchicus) from Eastern Poland: Distribution in Intestinal and Edible Tissue Samples and Species Identification by PCR and MALDI-TOF MS
Source: Foods. 2026 Sep 15;15(18):3262. doi: 10.3390/foods15183262 (PMC13606895; doi:10.3390/foods15183262)
Supplement: Supplementary file 1 [file foods-15-03262-s001.zip › Supplementary Figure S1.pdf]

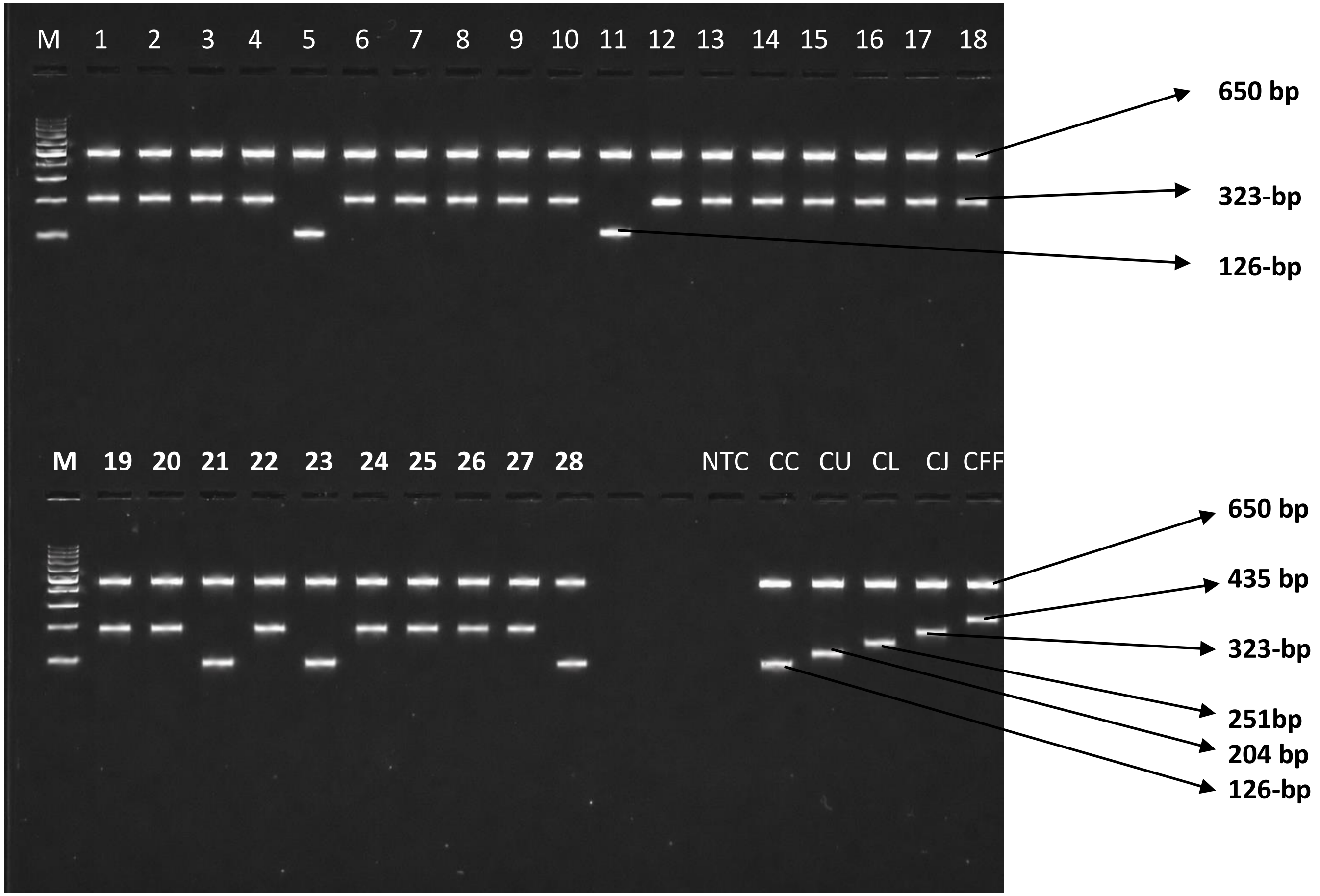

Figure S1. Multiplex PCR identification of *Campylobacter* isolates according to the method of Wang et al. [22]. M, 100-1000 bp DNA molecular size marker. Arrows indicate the expected sizes of the genus-associated 23S rRNA amplicon (650 bp) and species-specific amplification products. **Upper panel**, lanes 1-18: isolates 1-13FK, 2-13FW, 3-15FK, 4-16FP, 5-17FK, 6-32FK, 7-32FW, 8-33FK, 9-34FK, 10-34FW, 11-37FK, 12-38FK, 13-38FP, 14-39FP, 15-39FW, 16-40FK, 17-40FP, and 18-40FW, respectively. Isolates 17FK (lane 5) and 37FK (lane 11) were identified as *C. coli*, based on the presence of the 650-bp 23S rRNA amplicon and the species-specific 126-bp *glyA* amplicon. The remaining isolates in the upper panel were identified as *C. jejuni*, showing the 650-bp 23S rRNA amplicon together with the species-specific 323-bp *hipO* amplicon. **Lower panel**, lanes 19-28: isolates 19-50FK, 20-51FK, 21-53FK, 22-54FK, 23-56FK, 24-60FW, 25-61FW, 26-64FK, 27-64FP, and 28-70FK, respectively. Isolates 53FK (lane 21), 56FK (lane 23), and 70FK (lane 28) were identified as *C. coli*, whereas the remaining isolates were identified as *C. jejuni*. NTC, no-template control. CC, *C. coli* ATCC 33559™; CU, *C. upsaliensis* ATCC 43954™; CL, *C. lari* ATCC 35221™; CJ, *C. jejuni* ATCC 33560™; CFF, *C. fetus* subsp. *fetus* ATCC 27374™. The reference strains produced the expected species-specific amplicons of 126 bp (*C. coli*), 204 bp (*C. upsaliensis*), 251 bp (*C. lari*), 323 bp (*C. jejuni*), and 435 bp (*C. fetus* subsp. *fetus*), together with the 650-bp 23S rRNA amplification product.

Reference:

22. Wang, G.; Clark, C.G.; Taylor, T.M.; Pucknell, C.; Barton, C.; Price, L.; Woodward, D.L.; Rodgers, F.G. Colony multiplex PCR assay for identification and differentiation of Campylobacter jejuni, C. coli, C. lari, C. upsaliensis, and C. fetus subsp. fetus. *J. Clin. Microbiol.* **2002,** *40,* 4744–4747. https://doi.org/10.1128/JCM.40.12.4744-4747.2002.
