# Supplementary material for: Culture-Based Detection of Campylobacter spp. in Farmed and Free-Ranging Common Pheasants (Phasianus colchicus) from Eastern Poland: Distribution in Intestinal and Edible Tissue Samples and Species Identification by PCR and MALDI-TOF MS
Source: Foods. 2026 Sep 15;15(18):3262. doi: 10.3390/foods15183262 (PMC13606895; doi:10.3390/foods15183262)
Supplement: Supplementary file 1 [file foods-15-03262-s001.zip › Supplementary Table S5.pdf]

Table S5. Individual multiplex PCR and MALDI-TOF MS identification results for *Campylobacter* isolates recovered from farmed common pheasants.

Isolate ID	Bird ID	Sample matrix	Multiplex PCR identification	MALDI-TOF MS best match	Best-match score values (original report order)	Highest score	PCR–MALDI agreement
13FK	13F	Cloacal swab	*Campylobacter jejuni*	*Campylobacter jejuni*	2.111; 2.141; 2.254	2.254	Concordant
13FW	13F	Liver tissue	*Campylobacter jejuni*	*Campylobacter jejuni*	2.167; 2.171; 2.189	2.189	Concordant
15FK	15F	Cloacal swab	*Campylobacter jejuni*	*Campylobacter jejuni*	2.224; 2.167; 2.150	2.224	Concordant
16FP	16F	Pectoral muscle tissue	*Campylobacter jejuni*	*Campylobacter jejuni*	2.153; 2.035; 2.085	2.153	Concordant
17FK	17F	Cloacal swab	*Campylobacter coli*	*Campylobacter coli*	2.295; 2.320; 2.328	2.328	Concordant
32FK	32F	Cloacal swab	*Campylobacter jejuni*	*Campylobacter jejuni*	2.117; 2.114; 2.201	2.201	Concordant
32FW	32F	Liver tissue	*Campylobacter jejuni*	*Campylobacter jejuni*	2.178; 1.992; 2.187	2.187	Concordant
33FK	33F	Cloacal swab	*Campylobacter jejuni*	*Campylobacter jejuni*	2.210; 2.196; 2.156	2.210	Concordant
34FK	34F	Cloacal swab	*Campylobacter jejuni*	*Campylobacter jejuni*	2.308; 2.276; 2.245	2.308	Concordant
34FW	34F	Liver tissue	*Campylobacter jejuni*	*Campylobacter jejuni*	2.188; 2.249; 2.189	2.249	Concordant
37FK	37F	Cloacal swab	*Campylobacter coli*	*Campylobacter coli*	2.236; 2.211; 2.200	2.236	Concordant
38FK	38F	Cloacal swab	*Campylobacter jejuni*	*Campylobacter jejuni*	2.226; 2.275; 2.195	2.275	Concordant
38FP	38F	Pectoral muscle tissue	*Campylobacter jejuni*	*Campylobacter jejuni*	2.111; 2.122; 2.094	2.122	Concordant
39FP	39F	Pectoral muscle tissue	*Campylobacter jejuni*	*Campylobacter jejuni*	1.850; 2.059; 1.997	2.059	Concordant
39FW	39F	Liver tissue	*Campylobacter jejuni*	*Campylobacter jejuni*	2.121; 2.232; 2.194	2.232	Concordant
40FK	40F	Cloacal swab	*Campylobacter jejuni*	*Campylobacter jejuni*	2.062; 2.147; 2.028	2.147	Concordant
40FP	40F	Pectoral muscle tissue	*Campylobacter jejuni*	*Campylobacter jejuni*	2.109; 2.085; 2.039	2.109	Concordant
40FW	40F	Liver tissue	*Campylobacter jejuni*	*Campylobacter jejuni*	2.127; 2.155; 2.109	2.155	Concordant
50FK	50F	Cloacal swab	*Campylobacter jejuni*	*Campylobacter jejuni*	2.188; 2.131; 2.291	2.291	Concordant
51FK	51F	Cloacal swab	*Campylobacter jejuni*	*Campylobacter jejuni*	2.234; 2.268; 2.222	2.268	Concordant
53FK	53F	Cloacal swab	*Campylobacter coli*	*Campylobacter coli*	2.086; 2.335; 2.292	2.335	Concordant
54FK	54F	Cloacal swab	*Campylobacter jejuni*	*Campylobacter jejuni*	2.219; 2.237; 2.184	2.237	Concordant
56FK	56F	Cloacal swab	*Campylobacter coli*	*Campylobacter coli*	2.285; 2.309; 2.352	2.352	Concordant
60FW	60F	Liver tissue	*Campylobacter jejuni*	*Campylobacter jejuni*	2.009; 2.168; 2.181	2.181	Concordant
61FW	61F	Liver tissue	*Campylobacter jejuni*	*Campylobacter jejuni*	2.080; 1.947; 2.010	2.080	Concordant

Isolate ID	Bird ID	Sample matrix	Multiplex PCR identification	MALDI-TOF MS best match	Best-match score values (original report order)	Highest score	PCR–MALDI agreement
64FK	64F	Cloacal swab	*Campylobacter jejuni*	*Campylobacter jejuni*	2.210; 2.029; 2.036	2.210	Concordant
64FP	64F	Pectoral muscle tissue	*Campylobacter jejuni*	*Campylobacter jejuni*	2.101; 1.989; 2.163	2.163	Concordant
70FK	70F	Cloacal swab	*Campylobacter coli*	*Campylobacter coli*	2.116; 2.285; 2.189	2.285	Concordant

Bird IDs are presented using the numbering system applied in the supplementary dataset. K, cloacal swab; W, liver tissue; FP, pectoral muscle tissue. MALDI-TOF MS best-match scores are listed in the order in which they appeared in the original report. PCR–MALDI agreement was defined as assignment of the same species by multiplex PCR and MALDI-TOF MS.
