# Supplementary material for: Culture-Based Detection of Campylobacter spp. in Farmed and Free-Ranging Common Pheasants (Phasianus colchicus) from Eastern Poland: Distribution in Intestinal and Edible Tissue Samples and Species Identification by PCR and MALDI-TOF MS
Source: Foods. 2026 Sep 15;15(18):3262. doi: 10.3390/foods15183262 (PMC13606895; doi:10.3390/foods15183262)
Supplement: Supplementary file 1 [file foods-15-03262-s001.zip › Supplementary Tables S1-S4.pdf]

Table S1. Summary characteristics of the study population and sampling design.

Characteristic	Farmed Pheasants	Free-Ranging Pheasants	Total
Birds examined, n	71	83	154
Sampling locations, n	3 farms	11 hunting grounds	14 locations
Birds per location, n	20, 25, and 26	2-18	-
Females, n (%)	40 (56.3)	1 (1.2)	41 (26.6)
Males, n (%)	31 (43.7)	82 (98.8)	113 (73.4)
Age, months, mean ± SD	9.4 ± 2.9	14.4 ± 10.5	12.1 ± 8.4
Age, months, median (IQR)	10 (6-12)	10 (8-20)	10 (7-12)
Age, months, range	6-13	5-48	5-48
Sampling period	October 2022-January 2024	December 2022-February 2023	October 2022-January 2024
Birds with complete slaughter/hunting and laboratory examination records, n (%)	71 (100)	83 (100)	154 (100)
Interval from slaughter or hunting to laboratory examination, h, range	16-24	16-36	16-36
Samples collected per bird, n	4	4	4
Cloacal swabs examined, n	71	83	154
Caecal-content samples examined, n	71	83	154
Liver-tissue samples examined, n	71	83	154
Pectoral-muscle samples examined, n	71	83	154
Total samples examined, n	284	332	616

Table S2. Individual characteristics and sampling schedule of farmed common pheasants (*Phasianus colchicus*) (*n* = 71).

Bird ID	Sex	Age (Months)	Anonymized Origin	Slaughter Date	Laboratory Examination Date	Interval Range to Laboratory Examination (h)
1F	Female	6	Farm A	2022-10-09	2022-10-10	16-24
2F	Female	6	Farm A	2022-10-09	2022-10-10	16-24
3F	Male	6	Farm A	2022-10-09	2022-10-10	16-24
4F	Male	8	Farm A	2022-10-09	2022-10-10	16-24
5F	Female	6	Farm A	2022-10-09	2022-10-10	16-24
6F	Female	7	Farm A	2022-10-09	2022-10-10	16-24
7F	Female	6	Farm A	2022-10-09	2022-10-10	16-24
8F	Female	6	Farm A	2022-10-09	2022-10-10	16-24
9F	Female	7	Farm A	2022-10-09	2022-10-10	16-24
10F	Female	6	Farm A	2022-10-09	2022-10-10	16-24
11F	Female	12	Farm A	2023-05-14	2023-05-15	16-24
12F	Female	12	Farm A	2023-05-14	2023-05-15	16-24
13F	Female	12	Farm A	2023-05-14	2023-05-15	16-24

14F	Male	12	Farm A	2023-05-14	2023-05-15	16-24
15F	Female	12	Farm A	2023-05-14	2023-05-15	16-24
16F	Female	12	Farm A	2023-05-14	2023-05-15	16-24
17F	Female	12	Farm A	2023-05-14	2023-05-15	16-24
18F	Male	12	Farm A	2023-05-14	2023-05-15	16-24
19F	Female	12	Farm A	2023-05-14	2023-05-15	16-24
20F	Female	12	Farm A	2023-05-14	2023-05-15	16-24
21F	Female	12	Farm B	2023-05-14	2023-05-15	16-24
22F	Female	12	Farm B	2023-05-21	2023-05-22	16-24
23F	Female	12	Farm B	2023-05-21	2023-05-22	16-24
24F	Female	12	Farm B	2023-05-21	2023-05-22	16-24
25F	Female	12	Farm B	2023-05-21	2023-05-22	16-24
26F	Female	12	Farm B	2023-05-21	2023-05-22	16-24
27F	Female	12	Farm B	2023-05-21	2023-05-22	16-24
28F	Female	12	Farm B	2023-05-21	2023-05-22	16-24
29F	Female	12	Farm B	2023-05-21	2023-05-22	16-24
30F	Female	12	Farm B	2023-05-21	2023-05-22	16-24
31F	Female	12	Farm B	2023-05-21	2023-05-22	16-24
32F	Female	13	Farm B	2023-06-11	2023-06-12	16-24
33F	Female	13	Farm B	2023-06-11	2023-06-12	16-24
34F	Female	13	Farm B	2023-06-11	2023-06-12	16-24
35F	Female	13	Farm B	2023-06-11	2023-06-12	16-24
36F	Female	13	Farm C	2023-10-15	2023-10-16	16-24
37F	Female	13	Farm C	2023-10-15	2023-10-16	16-24
38F	Female	13	Farm C	2023-10-15	2023-10-16	16-24
39F	Female	13	Farm C	2023-10-15	2023-10-16	16-24
40F	Female	13	Farm C	2023-10-15	2023-10-16	16-24
41F	Female	13	Farm C	2023-10-15	2023-10-16	16-24
42F	Male	6	Farm C	2023-11-12	2023-11-13	16-24
43F	Male	6	Farm C	2023-11-12	2023-11-13	16-24
44F	Male	6	Farm C	2023-11-12	2023-11-13	16-24
45F	Male	6	Farm C	2023-11-12	2023-11-13	16-24
46F	Male	6	Farm C	2023-11-12	2023-11-13	16-24
47F	Male	6	Farm C	2023-11-12	2023-11-13	16-24
48F	Male	6	Farm C	2023-11-12	2023-11-13	16-24
49F	Male	6	Farm C	2023-11-12	2023-11-13	16-24
50F	Male	6	Farm C	2023-11-12	2023-11-13	16-24
51F	Male	6	Farm C	2023-11-12	2023-11-13	16-24

52F	Male	6	Farm B	2023-12-03	2023-12-04	16-24
53F	Male	6	Farm B	2023-12-03	2023-12-04	16-24
54F	Male	6	Farm B	2023-12-03	2023-12-04	16-24
55F	Male	6	Farm B	2023-12-03	2023-12-04	16-24
56F	Female	6	Farm B	2023-12-03	2023-12-04	16-24
57F	Female	6	Farm B	2023-12-03	2023-12-04	16-24
58F	Male	6	Farm B	2023-12-03	2023-12-04	16-24
59F	Male	6	Farm B	2023-12-03	2023-12-04	16-24
60F	Male	6	Farm B	2023-12-03	2023-12-04	16-24
61F	Male	6	Farm B	2023-12-03	2023-12-04	16-24
62F	Male	10	Farm C	2024-01-21	2024-01-22	16-24
63F	Male	10	Farm C	2024-01-21	2024-01-22	16-24
64F	Female	10	Farm C	2024-01-21	2024-01-22	16-24
65F	Male	10	Farm C	2024-01-21	2024-01-22	16-24
66F	Male	10	Farm C	2024-01-21	2024-01-22	16-24
67F	Male	10	Farm C	2024-01-21	2024-01-22	16-24
68F	Male	10	Farm C	2024-01-21	2024-01-22	16-24
69F	Male	10	Farm C	2024-01-21	2024-01-22	16-24
70F	Male	10	Farm C	2024-01-21	2024-01-22	16-24
71F	Male	10	Farm C	2024-01-21	2024-01-22	16-24

The recorded interval between slaughter and laboratory examination ranged from 16 to 24 h.

Table S3. Individual characteristics and sampling schedule of free-ranging common pheasants (*Phasianus colchicus*) (n = 83).

Bird ID	Sex	Estimated Age (Months)	Anonymized Origin	Hunting Date	Laboratory Examination Date	Interval Range to Laboratory Examination (h)
1W	Male	6	Hunting Ground 1	2022-12-17	2022-12-19	24-36
2W	Male	15	Hunting Ground 1	2022-12-17	2022-12-19	24-36
3W	Male	8	Hunting Ground 1	2022-12-17	2022-12-19	24-36
4W	Male	8	Hunting Ground 1	2022-12-17	2022-12-19	24-36
5W	Male	15	Hunting Ground 1	2022-12-18	2022-12-19	16-24
6W	Male	24	Hunting Ground 1	2022-12-18	2022-12-19	16-24
7W	Male	9	Hunting Ground 1	2022-12-18	2022-12-19	16-24
8W	Male	8	Hunting Ground 1	2022-12-18	2022-12-19	16-24
9W	Male	8	Hunting Ground 2	2023-01-08	2023-01-09	16-24
10W	Male	7	Hunting Ground 2	2023-01-08	2023-01-09	16-24
11W	Male	7	Hunting Ground 2	2023-01-08	2023-01-09	16-24
12W	Male	7	Hunting Ground 2	2023-01-08	2023-01-09	16-24

13W	Male	10	Hunting Ground 2	2023-01-08	2023-01-09	16-24
14W	Male	5	Hunting Ground 2	2023-01-08	2023-01-09	16-24
15W	Male	8	Hunting Ground 2	2023-01-08	2023-01-09	16-24
16W	Male	8	Hunting Ground 2	2023-01-08	2023-01-09	16-24
17W	Male	7	Hunting Ground 3	2023-01-15	2023-01-16	16-24
18W	Male	8	Hunting Ground 3	2023-01-15	2023-01-16	16-24
19W	Male	24	Hunting Ground 3	2023-01-15	2023-01-16	16-24
20W	Male	7	Hunting Ground 3	2023-01-15	2023-01-16	16-24
21W	Male	8	Hunting Ground 4	2023-01-22	2023-01-23	16-24
22W	Male	6	Hunting Ground 5	2023-01-22	2023-01-23	16-24
23W	Male	7	Hunting Ground 5	2023-01-22	2023-01-23	16-24
24W	Male	8	Hunting Ground 5	2023-01-22	2023-01-23	16-24
25W	Male	5	Hunting Ground 5	2023-01-22	2023-01-23	16-24
26W	Male	8	Hunting Ground 5	2023-01-22	2023-01-23	16-24
27W	Male	7	Hunting Ground 5	2023-01-22	2023-01-23	16-24
28W	Male	6	Hunting Ground 5	2023-01-22	2023-01-23	16-24
29W	Male	8	Hunting Ground 5	2023-01-22	2023-01-23	16-24
30W	Male	18	Hunting Ground 5	2023-01-22	2023-01-23	16-24
31W	Female	7	Hunting Ground 5	2023-01-22	2023-01-23	16-24
32W	Male	18	Hunting Ground 6	2023-01-29	2023-01-30	16-24
33W	Male	8	Hunting Ground 6	2023-01-29	2023-01-30	16-24
34W	Male	8	Hunting Ground 6	2023-01-29	2023-01-30	16-24
35W	Male	18	Hunting Ground 6	2023-01-29	2023-01-30	16-24
36W	Male	20	Hunting Ground 6	2023-01-29	2023-01-30	16-24
37W	Male	20	Hunting Ground 6	2023-01-29	2023-01-30	16-24
38W	Male	9	Hunting Ground 6	2023-01-29	2023-01-30	16-24
39W	Male	8	Hunting Ground 6	2023-01-29	2023-01-30	16-24
40W	Male	26	Hunting Ground 6	2023-01-29	2023-01-30	16-24
41W	Male	7	Hunting Ground 6	2023-01-29	2023-01-30	16-24
42W	Male	8	Hunting Ground 6	2023-01-29	2023-01-30	16-24
43W	Male	9	Hunting Ground 4	2023-02-05	2023-02-06	16-24
44W	Male	8	Hunting Ground 7	2023-02-05	2023-02-06	16-24
45W	Male	20	Hunting Ground 7	2023-02-05	2023-02-06	16-24
46W	Male	9	Hunting Ground 7	2023-02-05	2023-02-06	16-24
47W	Male	8	Hunting Ground 7	2023-02-05	2023-02-06	16-24
48W	Male	34	Hunting Ground 7	2023-02-05	2023-02-06	16-24
49W	Male	7	Hunting Ground 7	2023-02-05	2023-02-06	16-24
50W	Male	10	Hunting Ground 7	2023-02-05	2023-02-06	16-24

51W	Male	10	Hunting Ground 8	2023-02-12	2023-02-13	16-24
52W	Male	10	Hunting Ground 8	2023-02-12	2023-02-13	16-24
53W	Male	10	Hunting Ground 8	2023-02-12	2023-02-13	16-24
54W	Male	27	Hunting Ground 9	2023-02-12	2023-02-13	16-24
55W	Male	10	Hunting Ground 9	2023-02-12	2023-02-13	16-24
56W	Male	10	Hunting Ground 9	2023-02-12	2023-02-13	16-24
57W	Male	10	Hunting Ground 9	2023-02-12	2023-02-13	16-24
58W	Male	10	Hunting Ground 10	2023-02-12	2023-02-13	16-24
59W	Male	10	Hunting Ground 10	2023-02-12	2023-02-13	16-24
60W	Male	18	Hunting Ground 10	2023-02-12	2023-02-13	16-24
61W	Male	10	Hunting Ground 10	2023-02-12	2023-02-13	16-24
62W	Male	36	Hunting Ground 1	2023-02-19	2023-02-20	16-24
63W	Male	20	Hunting Ground 1	2023-02-19	2023-02-20	16-24
64W	Male	10	Hunting Ground 1	2023-02-19	2023-02-20	16-24
65W	Male	36	Hunting Ground 3	2023-02-19	2023-02-20	16-24
66W	Male	20	Hunting Ground 3	2023-02-19	2023-02-20	16-24
67W	Male	20	Hunting Ground 6	2023-02-19	2023-02-20	16-24
68W	Male	48	Hunting Ground 6	2023-02-19	2023-02-20	16-24
69W	Male	10	Hunting Ground 6	2023-02-19	2023-02-20	16-24
70W	Male	36	Hunting Ground 6	2023-02-19	2023-02-20	16-24
71W	Male	36	Hunting Ground 6	2023-02-19	2023-02-20	16-24
72W	Male	10	Hunting Ground 6	2023-02-19	2023-02-20	16-24
73W	Male	10	Hunting Ground 6	2023-02-19	2023-02-20	16-24
74W	Male	20	Hunting Ground 11	2023-02-19	2023-02-21	24-36
75W	Male	20	Hunting Ground 11	2023-02-19	2023-02-21	24-36
76W	Male	36	Hunting Ground 11	2023-02-19	2023-02-21	24-36
77W	Male	48	Hunting Ground 11	2023-02-19	2023-02-21	24-36
78W	Male	20	Hunting Ground 11	2023-02-19	2023-02-21	24-36
79W	Male	48	Hunting Ground 11	2023-02-19	2023-02-21	24-36
80W	Male	5	Hunting Ground 11	2023-02-19	2023-02-21	24-36
81W	Male	8	Hunting Ground 11	2023-02-19	2023-02-21	24-36
82W	Male	12	Hunting Ground 11	2023-02-19	2023-02-21	24-36
83W	Male	9	Hunting Ground 11	2023-02-19	2023-02-21	24-36

Age of free-ranging male pheasants was estimated in the field based on spur and plumage characteristics and should be interpreted as an approximate value rather than exact chronological age. The recorded interval between hunting and laboratory examination ranged from 16 to 36 h. For birds for which the recorded hunting and examination dates differed by two calendar days, the actual storage interval did not exceed 36 h.

Table S4. Primers and reaction concentrations used in the multiplex PCR assay for identification of *Campylobacter* isolates.

Target Species or Function	Target Gene	Primer Name	Sequence (5′–3′)	Amplicon Size (Bp)	Working Concentration (µm)	Volume per ReactIon (µl)	Final Concentration (µm)	Reference
Campylobacter jejuni	*hipO*	CJF	ACTTCTTTATTGCTTGCTGC	323	25	0.5	0.5	Wang et al. [22]
		CJR	GCCACAACAAGTAAAGAAGC	323	25	0.5	0.5	
Campylobacter coli	*glyA*	CCF	GTAAAACCAAAGCTTATCGTG	126	50	0.5	1.0	
		CCR	TCCAGCAATGTGTGCAATG	126	50	0.5	1.0	
Campylobacter lari	*glyA*	CLF	TAGAGAGATAGCAAAAGAGA	251	25	0.5	0.5	
		CLR	TACACATAATAATCCCACCC	251	25	0.5	0.5	
Campylobacter upsaliensis	*glyA*	CUF	AATTGAAACTCTTGCTATCC	204	100	0.5	2.0	
		CUR	TCATACATTTTACCCGAGCT	204	100	0.5	2.0	
Campylobacter fetus subsp. *fetus*	*sapB2*	CFF	GCAAATATAAATGTAAGCGGAGAG	435	50	0.5	1.0	
		CFR	TGCAGCGGCCCCACCTAT	435	50	0.5	1.0	
Internal validation control	23S rRNA	23SF	TATACCGGTAAGGAGTGCTGGAG	650	10	0.5	0.2	
		23SR	ATCAATTAACCTTCGAGCACCG	650	10	0.5	0.2	

Each PCR reaction was performed in a final volume of 25 µL containing 12.5 µL of 2× qPCR Master Mix, 0.5 µL of each of the 12 primers (6.0 µL total primer volume), 2.5 µL of genomic DNA, and 4.0 µL of nuclease-free water. All primers were prepared from 100 µM stock solutions. The 650-bp 23S rRNA amplicon generated by the 23SF/23SR primer pair served as an internal validation control, as described by Wang et al. [22].

Reference:

22. Wang, G.; Clark, C.G.; Taylor, T.M.; Pucknell, C.; Barton, C.; Price, L.; Woodward, D.L.; Rodgers, F.G. Colony multiplex PCR assay for identification and differentiation of *Campylobacter jejuni*, *C. coli*, *C. lari*, *C. upsaliensis*, and *C. fetus* subsp. *fetus*. *J. Clin. Microbiol.* **2002**, *40*, 4744–4747. https://doi.org/10.1128/JCM.40.12.4744-4747.2002.
